# From understanding to justifying: Computational reliabilism for AI-based forensic evidence evaluation

**DOI:** 10.1016/j.fsisyn.2024.100554

**Published:** 2024-08-30

**Authors:** Juan M. Durán, David van der Vloed, Arnout Ruifrok, Rolf J.F. Ypma

**Affiliations:** a Delft University of Technology, Jaffalaan 5, 2628 BX, Delft, Netherlands; b Division of digital and biometric traces, Netherlands Forensic Institute, Laan van Ypenburg 6, 2497 GB, Den Haag, Netherlands

**Keywords:** Forensic AI, Reliabilism, Explainability, Evidence evaluation

## Abstract

Techniques from artificial intelligence (AI) can be used in forensic evidence evaluation and are currently applied in biometric fields. However, it is generally not possible to fully understand how and why these algorithms reach their conclusions. Whether and how we should include such ‘black box’ algorithms in this crucial part of the criminal law system is an open question that has not only scientific but also ethical, legal, and philosophical angles. Ideally, the question should be debated by people with diverse backgrounds.

Here, we present a view on the question from the philosophy of science angle: computational reliabilism (CR). CR posits that we are justified in believing the output of an AI system, if we have grounds for believing its reliability. Under CR, these grounds are classified into ‘reliability indicators’ of three types: technical, scientific, and societal. This framework enables debates on the suitability of AI methods for forensic evidence evaluation that take a wider view than explainability and validation.

We argue that we are justified in believing the AI's output for forensic comparison of voices and forensic comparison of faces. Technical indicators include the validation of the AI algorithm in itself, validation of its application in the forensic setting, and case-based validation. Scientific indicators include the simple notion that we know faces and voices contain identifying information along with operationalizing well-established metrics and forensic practices. Societal indicators are the emerging scientific consensus on the use of these methods, as well as their application and interpretation by well-educated and certified practitioners. We expect expert witnesses to rely more on technical indicators to be justified in believing AIsystems, and triers-of-fact to rely more on societal indicators to believe the expert witness supported by the AIsystem.

## 1 Introduction

In criminal cases, expert witnesses give opinions on evidential value within the bounds of their expertise. The main justification for the trier of facts to believe this opinion is the credibility of the expert: demonstrable expertise and impartiality. In practice, experts explain their reasoning, e.g., point out the features of the evidence they took into account, to increase trust in their judgment further.

Increasingly, expert witnesses are aided by algorithmic methods grounded in forensic statistics. Such methods have the advantage that they can be validated and are more robust against human biases. For example, DNA experts utilize algorithms from probabilistic genotyping to obtain a numerical likelihood ratio (LR). The use of probabilistic genotyping is now widely accepted after a period of heavy discussion [1,2]. Part of this acceptance rests on the statistical model used being explainable: statisticians understand precisely what every parameter in the equation represents.

In most forensic fields, no statistical models exist that are both explainable and suitable to provide LRs. In some fields, notably biometrics, more complex deep learning models from the field of artificial intelligence (AI) are used. These have been shown to work very well in providing LRs, can be validated, and are less prone to biases than human experts. Given the rapid advances in deep learning, it is likely that more forensic fields will follow suit in employing such models in practice, particularly those interpreting images, audio, video, or text.

However, this class of AI models is not explainable in the sense that researchers cannot tell what the individual parameters of the model represent nor predict what the model would output for slightly perturbed input data. For this reason, the algorithms are often referred to as ‘black boxes’.^1^ Traditional statistical models like probabilistic genotyping are based on theory describing how the underlying system (e.g., population genetics) works. In contrast, these AI models are based on patterns that algorithms found in large amounts of data - with no guarantee they are the relevant patterns. The canonical example is a model that distinguished wolves from huskies on the presence of snow in the background [3], after training on a set that had wolves shown in the snow. The issue is that a) no human would make this mistake as it is clear to us that this does not generalize - huskies can be in snow as well, and b) it can be easy to miss that such a correlating feature as snow exists in the dataset and is used by the model. It is hard to see how something similar could happen for probabilistic genotyping.

There is debate in the AI literature and society as a whole on whether we should ‘trust’ such complex models [[4], [5], [6], [7]]. This is particularly pertinent in the forensic evidence evaluation setting, as the output can have a profound impact on people's lives. The question is very similar to those asked in the medical domain, where professionals make impactful decisions based on the outcome of complex models. It should be clear that this broad question of trust merits an interdisciplinary approach.

This article presents a perspective on AI-based evidence evaluation from the philosophical field: the “justification” option. The justification option consists of shifting unwarranted claims about explanation and understanding of the AI output to claims of having justification for believing it. Under the justification option, the forensic expert can indicate that the AI is part of a more extensive methodology that involves experts, that the methods involved have a high rate of success, and that the output of the AI is coherent with other similar results utilizing different methods, among a myriad of other indicators that secure justification. Thus, whereas forensic experts might still not be able to offer a full-blown explanation of how the system operates, they do have indicators that the entire process of producing outputs using AI is reliable, and therefore, they are well justified in believing that the output provides an appropriate and meaningful answer to the question posed in the context of the case. Within our viewpoint, being justified not only resolves the problems of explanation and understanding aforementioned, but also gives triers of fact a solid basis for including AI-based outputs as relevant components for the evidential base.^2^

With these ideas in mind, the article is divided into the following sections. Section 2 introduces our two motivating examples: forensic analysis of faces and voices. Section 3 introduces the aforementioned ‘justification option’. Finally, section 4 lays out the operationalization of this concept as three indicator types, and discusses how these apply to AI-based forensic evidence evaluation.

## 2 AI-based forensic evidence evaluation

We briefly discuss two forensic fields in which complex AI models are already being used in practice: face and speaker comparison. These are examples of a wider trend of using data-driven evidence evaluation [8,9].

### 2.1 Forensic face comparison

The question in forensic face comparison is whether the face seen in two images is from the same person. Often, one image is linked to a crime, e.g., a recording from a security camera, and the other image is a facial image of the suspect. The images may differ in quality (resolution, face angle, lighting), or there may be a long time period between the recording of the images. The question for the expert is ‘what would be the probability of observing the features of the facial images if they were of the same person relative to the probability of observing the features of the facial images if they were of two different people from the relevant population.’ The judgment of the expert is often expressed as a verbal conclusion.

According to the Facial Image Scientific Working Group (FISWG), morphological analysis is the primary approach used for forensic face comparison. This process involves a visual examination by experts, assessing the general shape of the face, as well as the shape, proportions, and relative position of facial features. The analysis extends to specific details, considering factors such as the presence of wrinkles, moles, scars, or tattoos, as well as the circumstances under which the face was imaged (pose, distance, lighting conditions, etc.). Each facial trait is evaluated by the experts according to an observation list, with the number of features and the degree of similarity and difference forming the basis for the final judgment.

In recent years, many tests have shown that deep learning models perform superiorly to humans in recognizing faces [10]. Deep learning-based automated forensic face comparison (AFFC) can be used by expert witnesses in criminal court cases [11]. Typically, the AFFC outputs a score that is a measure of the similarity between the two facial images. The evidential value of the score in the case can then be computed by considering the distribution of scores for same-person and different-person pairs of images that share recording characteristics with those in the case (e.g. lighting, pose, resolution). The characteristics of the facial images in the case at hand define the set of facial images of the alternative hypothesis and, thus, what facial images to include in the dataset. The dataset may be further determined by general features of the person observable in the questioned image (e.g., gender, demographics). The distribution of scores obtained for same-person facial image comparisons and different-person facial image comparisons then gives the evidential value of any given score as the ratio of their density [12]. The difficulty lies in knowing what recording characteristics are relevant and obtaining the representative dataset. Approaches to determine the characteristics of the images are using unsupervised AI-based generic quality estimation [13] or a facial image comparison method-specific quality analysis using Confusion Scores [12,14].

### 2.2 Forensic speaker comparison

Forensic speaker comparison is an examination in which a judgment is made on the speaker's identity in some recorded unknown voice by comparing it to a recording of the voice of a known speaker. The unknown voice is often linked to a crime and the known voice often belongs to the suspect of that crime. The recordings may vary in a large number of aspects, meaning that every case will have its own case conditions. Some of these are technical in nature, e.g., telephone/microphone, noise levels, reverberation, and some are connected to speaker behavior, e.g., spoken language, vocal effort, and health condition. If the likelihood ratio framework is used, the question the expert answers is ‘how much more likely is it to observe the speech features if the two recordings contain the same person (same-speaker hypothesis) than if the recordings contain two different people from the relevant population (different-speaker hypothesis)’. The expert needs to consider the case conditions when answering this question.

Most often, a human-based method is used [[15], [16], [17]]. This type of examination is performed by linguists who listen to the audio, noting salient speech properties and measuring acoustic properties. They will then use these properties to arrive at a conclusion regarding speaker identity while considering the case conditions. Over time, the performance of automatic speaker recognition (ASR) technology has increased to the extent that it can be leveraged for forensic casework, even outperforming lay people in some voice comparison tasks [[18], [19], [20]]. A growing number of forensic scientists now use such systems for forensic speaker comparison, often alongside the human-based method. Some reasons to include ASR in forensic speaker comparison are that it is not as sensitive to bias as humans are and that it is possible to measure the performance of the method with a large number of tests, which is infeasible for a human-based method.

In the application of current state-of-the-art ASR to forensic voice comparison, a set of features are derived from the audio. These features capture acoustic properties of the speaker's voice, but they also contain unwanted ‘channel information’, which depends on the case conditions. These features are then fed into a neural network that is trained with a large number of diverse speakers in all kinds of conditions. This training aims to allow the neural network to create speaker representations that retain speaker information and discard channel information as much as possible. The two speaker representations are entered into a statistical model that produces a score^3^. When the practitioner uses off-the-shelf software to do this, there is no access to the pre-training or ability to do additional pre-training. To make up for this, most software developers allow adapting the model using additional case-specific, user-submitted data.

Then, audio with known speaker identities in the conditions of the case are selected and formed into a number of same-speaker pairs and different speaker pairs, of which a part serves as calibration data and a part serves as validation data. The calibration data are used to calculate same-speaker scores and different-speaker scores. These are then used to train a calibration model. The calibration model is then used to calibrate scores resulting from the validation data and to calibrate the score obtained from the recordings in the case. The validation data are used to assess how well the forensic-speaker-comparison system performs under the conditions of the case.Both calibration data and validation data must be representative of the case data because otherwise, the calculated likelihood ratio and the validation results will not be meaningful. If all is well, a meaningful likelihood ratio is calculated, which is a measure of evidence strength and the method's end result. See for more on this [21].

ASR-based methods are more objective than the human method in the sense that the most important subjective steps in the latter are either completely left to the algorithm or substantiated by empirical research in the former. The actual comparison of features is left to the algorithm, and their interpretation of the end result is based on empirical data. However, the expert still has subjective judgments to make, the most important one being the choice of data that represent the case conditions.If some relevant condition is overlooked, the calibration data and validation data may differ from the case data in some systematic way. This would mean that the assessment of whether the algorithm was valid for the case conditions would be flawed. It would also mean that the computed likelihood ratio would have been calculated using the wrong data and would not be a meaningful answer to the question formed by the hypotheses for the case.This may result in a computed strength of evidence that is higher or lower than what would be obtained if the condition were not overlooked.

The end result of the method is an empirically based likelihood ratio, which is created in a repeatable way using a validated method.

## 3 Justification for believing

Is good performance, e.g., on a standard metric like accuracy, enough to have confidence in a system? A good counter-example is the 2016 study by Wu and Zhang, who presented a deep learning algorithm capable of inferring criminality from human facial traits [22,23]. This algorithm classifies individuals into two major categories: {criminals} and {non-criminals}, depending on the classification of an input photo under normal conditions of light, angle, and other variables. The authors reported high accuracy, approximately 95 % in most cases. Yet most people would agree that this empirical performance measurement in itself is not enough to justify a belief that criminality can be classified from faces. There is more than performance metrics.^4^ We argue that a more comprehensive perspective is given by ‘justification.’

We start with a simple definition of justification.

(1) A subject *S* is justified in believing a proposition *p* if *S* has reasons that ground or support the veracity of that *p.*^5^

We say we are justified in believing that *p* = “it is going to rain today” because we have reasons or evidence that support *p*. Our evidence could range from hearing the croaking of frogs (should you be lucky enough to live near an extension of water) to geo-spatial data collected and analyzed by the national weather service. While both support the belief that it is going to rain today, evidently, the latter provides stronger support. While it is not within our goals to discuss when and under what conditions one set of reasons or evidence is better than another –this point is further discussed below in the context of forensic AI– this issue does give us a purchase to consider *degrees* of justification. Typically, it is not a binary situation: it is not true that we are either justified or not justified to believe that it will rain. Instead, our support tends to be more nuanced: if the chances of rain are around 75 %, we take an umbrella with us. Our belief that *p* = “it is going to rain today” is partially justified by the estimated probability of rain. The degree of justification of a belief might shift again when new meteorological information comes to light. After carrying the umbrella with me all day, a southeast wind blew away the clouds expected to bring the rain. New information made my belief at a previous time unjustified. Bearing this in mind, we could say that.

(2) A subject *S* is *better* justified in believing *p* at time *t* if *S* has reasons that ground the veracity of *p* at time *t*.

Here we should point out that in forensic evidence evaluation, the proposition p is not simply one of the hypotheses (confusingly sometimes referred to as propositions in the forensic literature). Rather, it is the likelihood ratio itself, the level of support for the same-source vs the different source hypothesis. Thus, the question is whether a subject S is justified in trusting the value of the likelihood ratio - itself derived using an AI system.

There is a crucial distinction between justification, on the one hand, and explanation and understanding, on the other. As mentioned, justification involves having evidence or reasons that ground a given belief. As shown before, one can take the National Weather Services’ data as evidence supporting the belief that it will rain today. On the other hand, an explanation would involve showing how the National Weather Services processes geospatial data to render a probability of rain. Consumers of weather forecasts do not need to understand physics or meteorological computations; they have to trust experts who do.

The distinction between justification and explanation –and understanding– also applies mutatis mutandis to forensic AI. Using a reliable forensic speaker recognition system, for instance, the expert is better justified in believing that the output provides an appropriate and meaningful answer to the question posed in the context of the case. None of these particular epistemic maneuvers require explaining how the system generated the output or internalizing the principles and mechanisms underlying the algorithm. Rather, we care more about the practices, methods, data, and metrics used in and with forensic speaker recognition systems. If these are reliable, the system's outputs are too. Triers-of-fact do not need to understand the details of forensic evidence evaluation - they need to be able to trust the experts and the systems they use. Of course, the question now is what is a reliable forensic speaker recognition system? The next section will discuss this and other issues in more detail.

To finalize, let us quickly illustrate how justification is more successful in dismissing the AI utilized for inferring criminality based on facial traits than XAI [23]. Recall that, to explain how their AI classifies a given image as {criminal} or {not-criminal}, the expert needs to show how the algorithm classifies a given photo as {criminal} or {not-criminal}. This process typically involves highlighting key criteria by which the algorithm clusters photos, such as the distance between the eyes, curvature of the nose, and other facial traits. Since there is no particular problem with carrying out this explanation, the experts are, prima facie, in a position to make claims about explanation and understanding. However, despite the possibility of explanations, there is no scientific theory underlying the detection of criminal behavior from facial traits, as Wu and Zhang readily admitted. This is precisely the reason we do not feel justified in trusting their assertions, notwithstanding (limited) explainability and validation. The example illustrates that there are varied grounds for justifying our beliefs, all of which contribute to some extent. In the absence of one ground, we may still be justified in believing AI's output if the other grounds are strong.^6^ It is precisely this notion that computational reliabilism tries to make explicit.

## 4 Computational reliabilism

Justification to believe in the scientific value of an algorithm's output can be rooted in *computational reliabilism* (CR) [[24], [25], [26]]. Fundamentally, CR posits that outputs formed by reliable algorithmic processes are better justified than those formed by unreliable ones. In other words, we are better justified in trusting the outputs of an AI algorithm under the condition that the algorithm is reliable. An algorithm is considered reliable when it consistently generates scientifically valid outputs. Furthermore, CR operates under a probabilistic theory that tolerates occasional errors, misclassifications, and low predictive accuracy as long as, overall, the algorithm proves to be reliable—continuously producing outputs with scientific value.

The main issue for CR is to determine what makes an algorithm a reliable belief-forming process. CR deals with this issue by identifying different types of *reliability indicators* (RI), understood as algorithmic-related methods, standards, metrics, practices, and a wealth of knowledge inherent in the design, development, use, and maintenance of the algorithms and the data used. This idea will become clearer once we analyze each RI in some detail in the next sections. For now, the general idea is that each Type-RI can be understood as a marker for good methodological, scientific, and social practices, with the capacity to credit AI algorithms reliability. Here, we describe three such Type-RIs. As we will see in the next section, each Type-RI contains one or more Token-RI^7^ [26].

1. Type_1_-RI *Technical performance of algorithms* focuses on the design, coding, execution, maintenance, and other technical features that make a system perform well (e.g., in terms of robustness, precision, and accuracy). This includes the collection, curation, storage, distribution, and analysis of data; parametrizations; modularity; and other practices pertaining to algorithms.
2. Type_2_-RI *Computer-based scientific practice* focuses on AI-based scientific research. It results from the algorithmic implementation of common knowledge, scientific theories, principles, hypotheses, and other relevant units of scientific analysis. It also accounts for scientific interactions, debates, and other ways of engaging in scientific research that involve AI;
3. Type_3_-RI *Social construction of reliability* focuses on broader goals related to accepting AI and its outputs in diverse communities (e.g., scientific, academic, and public communities). This occurs through debate and similar forms of intellectual exchange. This type also includes social constructs such as regulatory bodies.

In what follows, we illustrate how computational reliabilism (CR) can be applied to forensic AI cases such as automatic speaker recognition applied to forensic voice comparison (ASR) and automatic forensic face comparison (AFFC) to justify the expert in trusting the scientific value of their outputs, and justify the trier-of-fact in trusting the output of the expert.

### 4.1 Type_1_-RI Technical performance of algorithms

Take Type_1_-RI, where reliability primarily arises from enhancing the robustness, precision, and accuracy of algorithms. Verification and validation methods (Token_1_-RI), encompassing various sub-categories (see, for instance, Oberkampf and Roy, 2010), exemplify approaches aligned with this goal. Achieving high accuracy and minimizing errors indisputably enhances the reliability of algorithms. Outputs also hinge on the user's comprehension of their scope, its suitability for the intended purpose, embedded assumptions, trade-offs made for tractability, and the algorithm's representative performance. For statistical evidence evaluation methods, calibration is another crucial characteristic to measure (i.e., ‘are the LRs of the right magnitude’) [27]. The clearest examples of Token_1_-RI are the full suite of performance metrics, such as log likelihood ratio cost (C_llr_), Tippet plot, equal error rate, etc. (for a more detailed treatment see e.g., Refs. [21,28]).

Another important Token_1_-RI are *redundancy mechanisms* designed to mitigate errors arising from unlucky executions of the algorithm or unlucky sampling of data. For instance, if there is enough case data, sampling multiple subsets of that data and performing the algorithmic comparison multiple times serves the purpose of minimizing the impact of random errors on the final result.

Possibly an even more important reliability indicator is the *representativeness of the data* - both the data used for construction and for testing of the algorithms. A key issue with many ASR systems and AFFC systems that undermines their reliability is that they have been verified against specific cases, *de facto* excluding others. For instance, if an ASR system has been trained and verified using only native English American accents, its performance in evaluating recordings from non-native English-speaking individuals is unknown, and thus its reliability considered low. This may not be a problem in a case involving only English American accents, but it will be otherwise. In other words, it is crucial that the validation data are representative of the application - the case at hand.

Such representativeness can relate both to characteristics of the person of interest (age, sex, accent) and to recording conditions (resolution, face angle, lighting, background noise, codec). The reliability of this indicator varies as a function of this representativeness. Data will never be fully matched. However, the more similar the data are, the more reason the expert has to believe that the output of the algorithm has scientific value.

The study by Wu and Zhang is a great example of the importance of using the right dataset for validation. The paper certainly presents efforts at validation, reporting an accuracy of 89.51 % (a standard Type_1_-RI indicator). But the photos they use for the {criminal} population are supplied by law enforcement authorities, whereas photos for {non-criminals} were found via a web crawl. This could lead to a very different distribution of recording conditions and subject expressions for an AI algorithm to pick up on. Furthermore, using convictions as ground truth for criminality is troublesome as racially biased policies or forced confessions play a role in many countries. Thus, Type_1_-RI indicators do not provide justification for believing in reading criminality from faces.

A more technical Token_1_-RI is the maturity of the software development process that gave rise to the algorithm. Were quality assurance methods used, such as well-known and tested libraries, code reviews, and version control? Although a convoluted script written under time pressure by a single person may perform well, we are more justified in trusting the output of software that has been constructed according to quality standards and can be checked by others.

A final Token_1_-RI worth mentioning is *robustness analysis*. A key virtue of this method is that it allows researchers to learn whether the performance of a given algorithm only holds for a narrowly defined dataset or whether they are likely to generalize. Thus, the algorithm should be tested on a wide set of possible data points, ideally including extremes. The core assumption is that if an algorithm performs well for a sufficiently heterogeneous set of data, then it is very likely that it will perform well in practice. Robustness analysis is a key indicator in the process of attributing reliability to AI algorithms. Note that this mainly applies to testing the base AI algorithm, e.g., a generic speaker comparison algorithm. When applied to casework, the relevant validation is on data representative of the case.

### 4.2 Type_2_-RI computer-based scientific practice

Type_2_-RI, on the other hand, directs attention to how scientific theories, hypotheses, principles, and other propositions grounded in the scientific discourse are operationalized into the algorithm or the databases used. It is noteworthy that such embedding may not always occur explicitly and intentionally. Researchers might not consciously operationalize a specific set of scientific propositions into the algorithm. Traditional statistical modeling may put much emphasis on formalizing scientific knowledge, while AI systems learn patterns from data and may thus not correspond to any scientific theory.

In simpler words, Type_2_-RI looks at whether algorithms are underpinned by (scientific) knowledge, theories, and principles. The lack of Type_2_-RI is the main reason we do not feel justified in trusting Wu and Zhang's face criminality inference algorithm. The idea that propensity to criminal behavior can be read from someone's face is part of long-discredited, pseudoscientific theories such as phrenology. Thus, even though their algorithm purportedly achieves high accuracy (a Type_1_-RI), we remain unconvinced of its reliability - we do not expect the output (criminality) to be obtainable from the input (faces). In a sense, these indicators capture our prior belief that the task the system purports to carry out is possible.

Both ASR and AFFC are based on a sound theoretical basis: faces and voices allow for the identification of people. This is, in fact, so obvious in everyday life that we generally don't view it as a scientific theory but a commonly known fact. However, knowing that the output can be constructed from the input is a crucial aspect for justifying the acceptance of the AI-based system's output. It is exactly the lack of this link that leads us to reject Wu and Zhang's face criminality algorithm.

### 4.3 Type_3_-RI Social construction of reliability

In the dynamic landscape of scientific inquiry, the role of social debate cannot be overstated. The social construct of reliability (trust) in the evaluation of forensic evidence involves the trust of the expert in the system used as well as the trust of the judge in the expert's viewpoint. Beyond the confines of epistemic security delivered by Type_1_-RI (e.g., low log likelihood ratio cost) and Type_2_-RI (the operationalization of scientific knowledge), the broader social sphere brings numerous benefits to the forensic community and society at large. The integration of AI into forensic applications, such as ASR and AFFC, necessitates robust scientific debate that strongly grounds the belief that the output of algorithms has forensic value. The Type_3_-RI, social construction of reliability, emphasizes the importance of active involvement and intellectual exchange in scrutinizing the outputs of forensic AI within diverse communities, including scientific, academic, and legal circles. These reliability indicators are grounded in legal doctrine, as most jurisdictions demand that forensic methodology employed should be accepted by the wider scientific community.

In order to train a statistical model that calculates or calibrates a likelihood ratio, a forensic practitioner has to obtain data that are representative of the relevant population and reflective of the conditions for the case. It is never the case that the expert assessment is taken at face value. Instead, forensic experts engage in discussions about recording conditions, the relevance of specific speaker characteristics, and the limitations inherent in their methods. Naturally, some constraints need to be set in place to avoid influencing each other, such as debating conditions of assessment after said assessment took place. Regardless, these debates are essential for increasing the forensic expert's belief in their assessments, including ensuring that they meet the stringent standards required in legal contexts. A similar social construction of justification can be thought of for ASR and AFFC algorithms.

Indeed, to justify their outputs, forensic experts undergo extensive discussions about the models and hyperparameters used, the values they are set to, as well as the applicability of the output and scientific soundness. Apart from this, they also need to be able to explain choices made in obtaining the result of the AI algorithm to the judge to establish trust in their judgment. For example, if an expert arrives at a certain likelihood ratio but has failed to use female-only recordings when the recordings in the case are clearly of females, other forensic experts are best placed to raise concerns and contest the output on scientific grounds. The same holds for AFFC, for example when the lighting conditions of a photo negatively affect the assessment given by an AFFC system. Note that being able to fully explain the workings of an algorithm would fall under this RI and, if possible, would certainly increase our justification for believing its output.

Type_3_-RI indicators are highly relevant for triers-of-fact as they are the most accessible of the three types. For example, whether a method has been independently scrutinized can be seen from publication in a peer-reviewed journal. Whether debates have been held and found resolved can be seen from acceptance in specific forensic fields and forensic science as a whole. Whether the method is mature, broadly adopted, and applied in the standard way is indicated by the existence and adherence to standards. Whether an expert is competent can be learned from certification. It is no coincidence that legal doctrine in many jurisdictions places much emphasis on these aspects. For example, in addition to empirical testing of a method, the Daubert ruling lists, as indicia of scientific validity, general acceptance of the method in the scientific field to which it belongs, existence of standards regulating the use of the method, and peer-reviewed publication of the method.

To illustrate the value of forensic type_3_-RI indicators, consider once again the deep learning algorithm inferring criminality based on facial traits [22]. As discussed, this algorithm classifies individuals into two major categories: {criminals} and {non-criminals}, depending on an input photo. The authors report a high predictive accuracy of approximately 90 %. Although the methodological flaws in the study may not be immediately obvious to all, the fact that it was neither peer-reviewed nor generally accepted in the field is relatively easy to ascertain.

Scientific debates around AI-based forensic evidence evaluation should extend beyond traditional scientific considerations. They involve interdisciplinary collaboration, incorporating expertise from computer science, law, statistics, and forensic science. These diverse views are needed to understand how well the algorithms perform in general, how they perform in the context of specific cases, how this can be optimally evaluated and communicated, and what these results mean for the legal requirements on evidence and expert witnesses in a given jurisdiction.

The established theory in this domain is exemplified by the fact that scientific debates in forensic AI are not limited to the algorithmic aspects alone. They delve into the broader context, encompassing legal, ethical, and societal considerations. For instance, debates may arise regarding the impact of facial recognition technology on civil liberties and the potential for bias in ASR outputs in forensic voice comparison based on cultural or linguistic factors.

The value of scientific debate in assessing forensic AI outputs, particularly in forensic comparisons using ASR and AFFC, cannot be overstated. The Type_3_-RI social construction of reliability emphasizes the need for active involvement and intellectual exchange to ensure the reliability of these technologies in diverse communities and, therefore, the justification of the expert's and trier-of-fact's belief in their outputs.

## 5 The limits of CR

At this point, it is important to highlight that CR represents a return to established scientific methodologies and practices, albeit with a unique twist. Now, researchers are compelled to integrate well-accepted principles of algorithmic design, utilization, and maintenance. According to CR, this integration enhances researchers' confidence in AI systems, justifying their belief in the scientific merit of the outputs and ultimately fostering the reliability of AI. Remarkably, all of this can be achieved without opening the black box.

Let us clarify that CR does not assert that, under its framework, AI systems are universally efficient, error-free, or suitable for all purposes. Additionally, CR acknowledges the human cognitive limitations in accessing some Token-RI, recognizing that reliability indicators are neither absolute nor universally applicable. Consequently, not all Token-RI are credited as reliable under the same criteria, and the same Token-RI does not apply with equal force to all AI algorithms.

We have defended the justificatory role of computational reliabilism. We have also defended the claim that justification is better suited for cases in forensic AI than explanation and understanding. Unfortunately, CR has some pitfalls that need to be addressed. In what follows, we briefly mention three issues that threaten to hamper CR's justificatory value and how to solve them. These are 1) the availability of each Token-RI; 2) that only a few indicators could determine the reliability of the system; and 3) establishing the weight of each Token-RI to the effect of justifying the output of an algorithm. Let us briefly address each one in turn.

The first limitation of CR is associated with the availability of any given reliability indicator. In such cases, researchers are tasked with evaluating the reliability of their system based on a limited set of indicators. For instance, it might be the case that the ASR algorithm utilized is proprietary, and therefore, experts are limited in their knowledge of how certain key concepts have been implemented (e.g., feature selection, initial training process). A way around this problem is to put more emphasis on Type_3_-RI *Social construction of reliability* where experts are in the loop, in the sense that they have a specific weight in the final decision.

Related to the limited availability of reliability indicators is that only a few of such indicators could decide on the reliability of an AI algorithm. Indeed, either due to the scarcity of indicators or because weights on each indicator have not been well distributed, a few indicators could have a disproportionate influence over the attributed reliability of the forensic AI. To illustrate this counterfactually, our assessment of the reliability of a system would most likely differ had we had access to all the relevant indicators. We term this phenomenon the *tyranny of the few*, underscoring the importance of having as many and as diverse reliability indicators as possible available.

Finally, it would be desirable to measure the reliability of a forensic AI. In this way, experts have a scale to determine how reliable a system is –in absolute or relative to another competing system. But this is a difficult matter in general, as the diverse Token-RIs are hard to quantify, and there is no objective way to decide on their relative importance. Different people may prefer a) a system that shows better accuracy overall, b) a system that has access to possibly more representative data or c) that has been scrutinized better in the literature. Depending on these preferences, the level of justification of the output might shift.

## 6 Discussions

The aim of forensic evidence evaluation is to state the degree to which a particular object, trace, or mark supports one hypothesis relative to another. A reliable realization of this aim is desirable not only for the public perception and trust in the judicial system but also because a person's future might well depend on a correct assessment.

Whether one is justified in trusting a system is, in the end, a subjective decision, which will be influenced by the decision maker's background. In forensic evidence evaluation, two such decisions take place. First, the expert witness has to decide whether they are justified in believing the algorithm's output. Given the technical background of the expert, most focus will be on technical RI_1_ and scientific RI_2_ indicators. Second, the trier-of-fact should decide in court whether they are justified in believing the experts' conclusions. Although the existence of RI_1_ indicators will play a large role for them, their nuances are hard to grasp for non-technical decision-makers. RI_3_ indicators will play the largest role - they are justified in believing the expert's output as the expert has the necessary qualifications, the proper accreditations are in place, and the scientific community has debated and largely agreed on the validity of the method used. Note that although we focused on AI-based evidence evaluation, the framework for justification is very general and certainly extends to other forms of algorithm-based evidence evaluation.

One important topic we did not touch upon is specific legal criteria around the use of evidence. Whether we are justified in believing the evidence evaluation will clearly be relevant in every jurisdiction, but never the sole consideration. We believe the conceptual framework offered by CR will be relevant in any jurisdiction, but clearly, the framework will not answer all relevant legal questions. For example, the question has been asked whether the right to a fair trial enshrined by the European Convention of Human Rights necessitates explainable methods [29], which would make our argument void. This example again underlines the importance of an interdisciplinary approach. Legal expertise is needed to understand the precise legal requirements, academic and data science expertise is needed to understand whether and how algorithms can fulfill such requirements, and forensic science expertise is needed to answer what solutions are feasible in practice.

Within this background, CR construes justification as inherently provisional but also as our best epistemic and cognitive efforts given context, restricted resources, and technological limits. In this respect, reliability is built from diverse self-monitoring, self-critical, and self-correcting scientific activities that constitute our best knowledge and practices, each subject to further revision and scrutiny. It is this whole set of indicators taken together that should guide our decision on whether we are justified in believing some output, rather than a single aspect (such as explanations).

## Funding

The research reported in this work was partially supported by the EU H2020 ICT48 project “Humane AI Net” under contract # 952026. The support is gratefully acknowledged. This work was supported by the European Commission through the H2020-INFRAIA-2018-2020/H2020-INFRAIA-2019-1 European project “SoBigData++: European Integrated Infrastructure for Social Mining and Big Data Analytics” (Grant Agreement 871042). The funders had no role in developing the research and writing the manuscript.

## CRediT authorship contribution statement

**Juan M. Durán:** Writing – review & editing, Writing – original draft, Investigation. **David van der Vloed:** Writing – review & editing, Writing – original draft, Investigation. **Arnout Ruifrok:** Writing – review & editing, Writing – original draft, Investigation. **Rolf J.F. Ypma:** Writing – review & editing, Writing – original draft, Investigation.

## Declaration of competing interest

The authors declare the following financial interests/personal relationships which may be considered as potential competing interests:

Juan M. Duran reports financial support was provided by 10.13039/501100001831Delft University of Technology. David van der Vloed reports administrative support was provided by Netherlands Forensic Institute. Arnout Ruifrok reports administrative support was provided by Netherlands Forensic Institute. Rolf J.F. Ypma reports administrative support was provided by Netherlands Forensic Institute. If there are other authors, they declare that they have no known competing financial interests or personal relationships that could have appeared to influence the work reported in this paper.

## Footnotes

5 For simplicity, we talk about “justification as giving reasons”, but more extensively, justification can be taken as providing reasons, evidence, or logical support to establish the validity, correctness, or appropriateness of a belief, action, or decision. Similarly, we take beliefs to be epistemic, but other forms of beliefs are possible such as moral, societal, legal, or political. For instance, to epistemically believe that it will rain today means that we have reasons, evidence, or the belief is logically supported. Contrast this with the fact that there is no moral stand in the belief that it will rain today, since a moral belief would amount to having reasons –evidence, etc.– that an action is good or bad for some purpose.

## Footnotes

1 Note that any algorithm can be a black box to some but not others, e.g. when the algorithm is not disclosed for proprietary reasons. In contrast, complex AI algorithms are a black box to all.

2 For a similar solution for the medical field, see Ref. [30].

3 Sometimes referred to as an uncalibrated likelihood ratio because it expresses not only similarity but also typicality

4 We will get back later to various flaws of the study later, which are helpful to understand different aspects of justification.

6 Of course, some grounds may be indispensable. As PCAST put it: “For forensic feature-comparison methods, establishing foundational validity based on empirical evidence is thus a sine qua non. Nothing can substitute for it."

7 A rough distinction between Type-RI and Token-RI is that while the former refers to the unique category of a family of reliability indicators, the latter refers to an individual occurrence of them. For instance, Type_1_-RI refers to any methodology, metric and practice that make the algorithm as a whole reliable. Any one individual, such as verification and validation techniques, qualify as Token_1_-RI.
